# Grapefruit Seed Extract-Added Functional Films and Coating for Active Packaging Applications: A Review

**DOI:** 10.3390/molecules28020730

**Published:** 2023-01-11

**Authors:** Swarup Roy, Wanli Zhang, Deblina Biswas, Rejish Ramakrishnan, Jong-Whan Rhim

**Affiliations:** 1 School of Bioengineering and Food Technology, Shoolini University, Solan 173229, India; 2 College of Food Science and Engineering, Hainan University, Haikou 570228, China; 3 Department of Printing Technology, College of Engineering Guindy, Anna University, Chennai 600025, India; 4 Department of Food and Nutrition, BioNanocomposite Research Center, Kyung Hee University, 26 Kyungheedae-Ro, Dongdaemun-gu, Seoul 02447, Republic of Korea

**Keywords:** GSE, films and coatings, antimicrobial, antioxidant, active packaging, food preservation

## Abstract

Recently, consumers have been increasingly inclined towards natural antimicrobials and antioxidants in food processing and packaging. Several bioactive compounds have originated from natural sources, and among them, grapefruit seed extract (GSE) is widely accepted and generally safe to use in food. GSE is a very commonly used antimicrobial in food; lately, it has also been found very effective as a coating material or in edible packaging films. A lot of recent work reports the use of GSE in food packaging applications to ensure food quality and safety; therefore, this work intended to provide an up-to-date review of GSE-based packaging. This review discusses GSE, its extraction methods, and their use in manufacturing food packaging film/coatings. Various physical and functional properties of GSE-added film were also discussed. This review also provides the food preservation application of GSE-incorporated film and coating. Lastly, the opportunities, challenges, and perspectives in the GSE-added packaging film/coating are also debated.

## 1. Introduction

Recently, food safety and security have been seriously recognized due to the growing problem of foodborne diseases worldwide. Food poisoning is often caused due to the growth of unwanted foodborne pathogenic microbes during food storage. Interestingly, pathogen proliferation sometimes occurs without changing the foods’ physical characteristics (color, flavor, and odor) [1,2]. Thus, reducing the risk associated with foodborne pathogens is very important. In this regard, food packaging or using food preservatives directly in the food or as a packaging matrix are traditionally used. Foodborne pathogens and food spoilage are generally controlled by employing food preservatives during food packaging. Food packaging films or coatings can improve the food shelf life and ensure food safety by limiting the growth of foodborne microbes [3,4,5,6,7]. Recently, functional food packaging is emerging as a hot topic to strengthen the safety and security of food.

**Figure 1:**
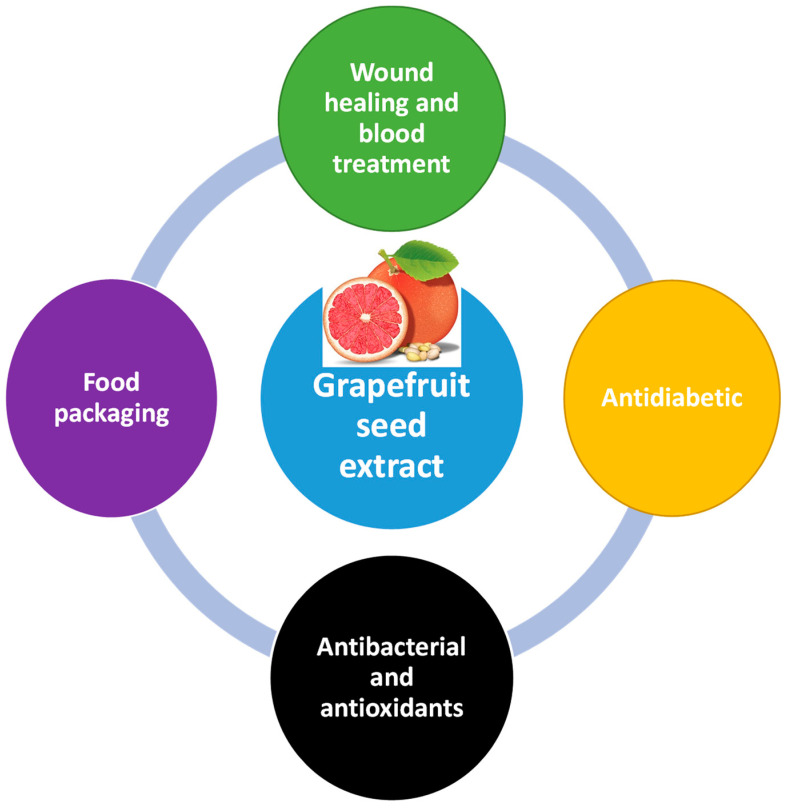
Biological properties and food applications of GSE.

In functional packaging, the antimicrobial and antioxidant compounds are infused into the packaging matrix. Commercially, countless chemical compounds are used as food preservatives. Even in the food packaging sector, many chemical compounds, like benzoic acid, propionic acid, sorbic acid, etc., are frequently used [8]. Since chemical preservatives are toxic, natural, or naturally derived, functional compounds are gaining popularity among consumers. To this end, essential oils and plant extracts have increased significantly recently due to their strong antibacterial and antioxidant activity and safety [4,9,10,11,12]. Among the various types of natural functional materials, grapefruit seed extract (GSE) is well known and broadly used in the food and pharmaceutical sector [1,13]. The application of GSE in health and food is represented in Figure 1, which clearly illustrates the potential of GSE [14]. GSE has been used in many applications such as wound healing, antioxidant, antibacterial, antidiabetic, preservative, food packaging, etc. [14]. 

GSE is citrus-derived functional material containing different bioactive components like flavonoids, polyphenols, organic acid, etc., which are believed to be responsible for the strong antimicrobial and antioxidant activity [1,15,16,17]. The strong functional properties of GSE have recently been used in combination with xylitol as a nasal spray to cure COVID-19 patients [18]. The strong antimicrobial activity of GSE has become a topic of debate, since it has been shown that commercial GSE includes some chemical csompounds, such as benzalkonium and benzethonium compounds, which shows antimicrobial activity [19,20]. Nevertheless, it has also been reported that the pure ethanolic extract of GSE showed potent antimicrobial activity toward foodborne pathogens [21]. Due to its strong functional properties, recently, there is growing interest in GSE-added film and coatings. GSE has been added to the varieties type of polymer matrices, such as alginate, agar, polylactic acid, carrageenan, gelatin, etc., to manufacture functional packaging film or coating [22,23,24,25,26,27,28]. Some of the reported functional films were used for real-time packaging of bread, fruits, or fish, and in most cases, it has been reported that the presence of GSE significantly improves the shelf life of packed food [29,30,31,32,33]. GSE-added packaging was highly effective in preserving fresh and processed food [26,29,34,35]. Even though plenty of research has already been published on GSE-added packaging films and coatings, there is no comprehensive review on this topic.

Therefore, this study aims to provide an update on the latest progress of GSE and its impact and use in food packaging film/coating applications. This review briefly discusses the GSE and its extraction and then summarizes the effect of GSE on the various physical and functional properties of the active food packaging film. In addition, we emphasize the application of GSE-added packaging films or coatings for fresh or processed food preservation.

## 2. Chemical Composition of Grapefruit Seed Extract

Grapefruit is a citrus fruit, and its seeds contain chemical composition, mainly, fixed oils, tocopherols, phytosterols, polyphenols, carotenoids, and minerals. The chemical composition of grapefruit seeds from different varieties and geographical sources is not quite the same. According to previous reports, the oil content of grapefruit seeds is about 40.2 to 45.5 %, similar to that of other citrus fruit seeds, such as Egyptian orange, mandarin, and lime [14,15]. Among them, the lipid fraction in grapefruit seeds consists mainly of both saturated and unsaturated or omega fatty acids, with palmitic acid (16:0), oleic acid (an omega-9 fatty acid, 18:1), and linoleic acid (an omega-6 fatty acid, 18:2) as major constituents amounting for more than 20 % in most grapefruit seed oils [15,36]. The light petroleum ether extracts of West Indian grapefruits seeds and other citrus fruit seeds exhibited similar fatty acid profiles mainly composed of palmitic (23–25%), stearic (5–10%), linoleic (nearly 40%), and oleic acid (20–25%) [37]. In addition, grapefruit seeds contain fat-soluble bioactive compounds such as tocopherols, carotenoids, and phytosterols. The tocopherol content of grapefruit seeds was reported to be 380 mg/kg [38]. 

The most bioactive compounds in GSE are phenolic compounds, including phenolic acids, i.e., trans-ferulic acid, rosmarinic acid, and trans-2-hydroxycinnamic acid, and flavonoids, as the main antioxidant active ingredient [15]. However, information on the detailed phenolic profiles in GSE is very limited. The reason may be that commercial GSE has been widely used in food and other industries, and the natural composition of commercial GSE is unreliable. Most studies in recent years have detected synthetic compounds from commercial GSE, such as benzethonium chloride, methylparaben, and benzalkonium chloride, and the contaminated synthetic compounds may be the main antimicrobial substance in commercial GSE [39]. Takeoka et al. (2001, 2005) performed compositional analyses of commercial GSE using a variety of instruments and methods, including HPLC, electrospray ionization mass spectrometry (ESI/MS), nuclear magnetic resonance (NMR) spectroscopy, and elemental analysis by proton-induced X-ray emission (PIXE) [20,40]. They showed that the main components of commercial GSE are benzethonium chloride and benzalkonium chloride. Similarly, in other studies, the main component in commercial GSE determined using the HPLC and quantitative NMR methods, respectively, also was benzethonium chloride [41,42]. However, another explanation for the occurrence of synthetic antimicrobial compounds in commercial GSE is the conversion of unstable polyphenols in GSE to quaternary ammonium compounds during the extraction and purification of GSE, such as benzethonium chloride and benzalkonium chloride [1]. 

Even though GSE contains antimicrobial quaternary compounds, it is thought that those compounds, rather than polyphenols exhibit antibacterial activity. Therefore, although numerous studies can determine that the main antimicrobial component of GSE, which is widely used in the food industry, is benzethonium chloride, the naturalness of benzethonium chloride is still more controversial [1,39]. In addition, GSE contains the characteristic compound limonoids, which belong to the oxygenated triterpenoids and is the main contributor to the bitterness of citrus fruits [43]. Limonoids, a characteristic compound of citrus fruit seeds, can be used to identify the quality and naturalness of GSE [20]. A related study has shown that GSE also contains furanocoumarins, such as bergamotin, epoxybergamotin, and 6′,7′-dihydroxybergamotin [44].

## 3. Extraction and Purification of GSE

**Figure 2:**
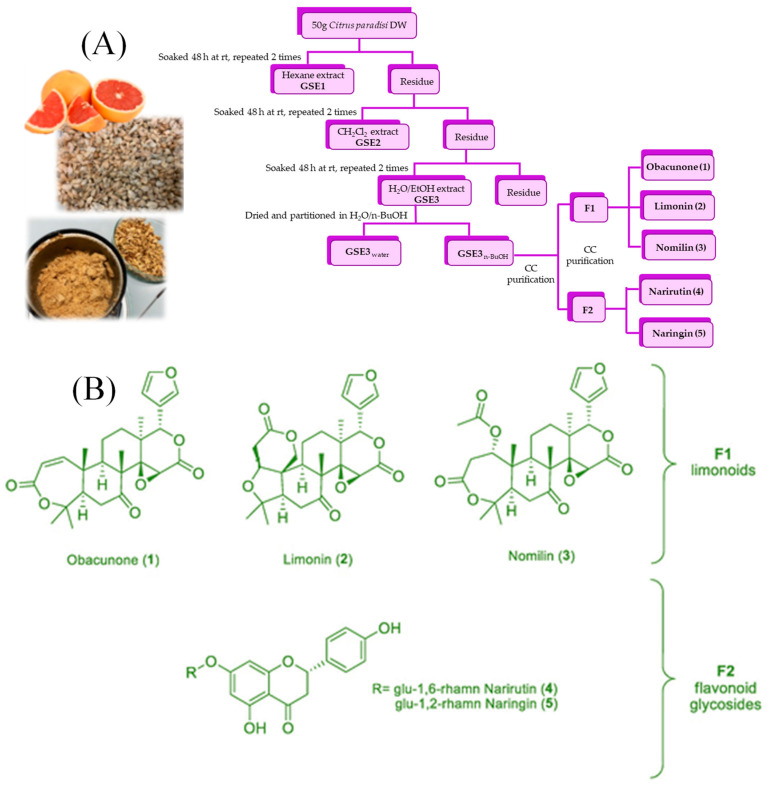
Extraction process of grapefruit seed extract (**A**), the structure of compounds extracted (**B**). (Adapted from Magurano et al., 2021) [46].

The extraction process of GSE is usually carried out in the powder state. The extraction process of GSE starts with collecting grapefruit seed, followed by washing, drying, and grinding to obtain grapefruit seed powder using a pulverizer. Similar to the extraction procedure of other plant polyphenol extracts, the extraction of active ingredients such as phenolics from grapefruit seeds in high yield requires optimization of several key factors, mainly, the degree of comminution, solvent type, extraction time, extraction temperature, and extraction pressure, etc. [15]. Extraction methods of plant polyphenols are generally divided into traditional and new extraction methods. Among them, traditional extraction methods are mainly based on liquid–liquid extraction methods, and the extraction solvents are usually ethanol, methanol, acetone, or formic acid and water in different proportions. The traditional methods include maceration, decoction, organic solvent, reflux extraction, etc. It was reported that pure ethanol solvent was more effective than water and other solvent mixtures in extracting the total polyphenol and flavonoid fractions in GSE [45]. The extraction process and structure of purified bioactive components are schematically presented in Figure 2. 

GSE extraction is straightforward as the residue can be extracted with ethanol/water after removing fatty and other substances. The major purified compounds analyzed using HPLC in GSE were limonin, obacunone, naringin, nomilin, etc. [46]. These methods often have poor extraction effects, low content of target components, many impurities, affect the efficacy of the medicine, and other disadvantages. New extraction methods include the ultrasonic-assisted extraction method, microwave-assisted extraction method, enzyme-assisted extraction method, and supercritical fluid extraction method. Compared with traditional extraction methods, these new technologies have advantages such as high purity of target components, high yield, and being energy saving [47]. In recent years, numerous studies have focused on applying environmentally friendly and efficient emerging technologies for the extraction and purification of GSE. For example, supercritical fluid extraction has been used to extract limonin glycosides and naringin derivatives from grapefruit seeds [48]. 

## 4. GSE-Added Functional Packaging Film

GSE contains a high concentration of citric acid, polyphenols, flavonoids, tocopherol, and ascorbic acids. Flavonoids derived from GSE are powerful antioxidants. Additionally, GSE has significant antibacterial action against Gram-positive and Gram-negative microorganisms. The main bioactive flavanone compound in GSE is naringin in large amounts. On the other hand, the flavanols hesperidin and quercetin exist in low concentrations. Studies related to GSE suggest that it can prevent the growth of foodborne pathogens in various fruits, vegetables, meat, and fish products [1]. GSE is commonly used as an antimicrobial agent in food packaging systems. Incorporating small quantities of GSE in various polymer solutions and coatings can induce their high microbial resistance and antioxidant properties, increasing the shelf life of various postharvest food products. GSE is thermally stable due to the presence of polyphenolic components, allowing it to endure the high processing temperatures used in food packaging materials and the thermal processing of packaged products. Recently, few reports on GSE include packaging films or coatings for active food packaging applications [1]. 

The solution casting method is most commonly used for GSE-added packaging film. The solvent casting method is the most widely used and easiest form of film formation, mostly used on a laboratory scale. The process follows the preparation of a dispersion/solution of a suitable solvent and later spreading on a surface and allowing it to dry the film by removing the solvent part. The prepared film’s mechanical, barrier, and surface properties will be influenced by parameters such as processing time, temperature, and drying conditions. The rapid drying reduces the polymer mobility during the drying process, affecting the intermolecular interactions. In film manufacturing methods, the solution casting technique has been widely used in preparing packaging materials at a laboratory scale because of its ease and simplicity. However, the solution casting technique is normally not practicable for commercial film production. This type of film production is difficult to scale up, and it includes several steps (solubilization, casting, and drying), which causes a relatively long processing time. 

On the contrary, the extrusion method allows for continuous operation with precise control over the temperature, size, shape, and moisture during the processing. Compared to the solution casting technique, the extrusion technique provides a more structured film and allows better dispersion of active compounds in the polymers. Therefore, the extrusion technique is usually preferred in industrial applications. Nevertheless, few studies have been on the preparation of biodegradable antimicrobial films using extrusion.

**Figure 3:**
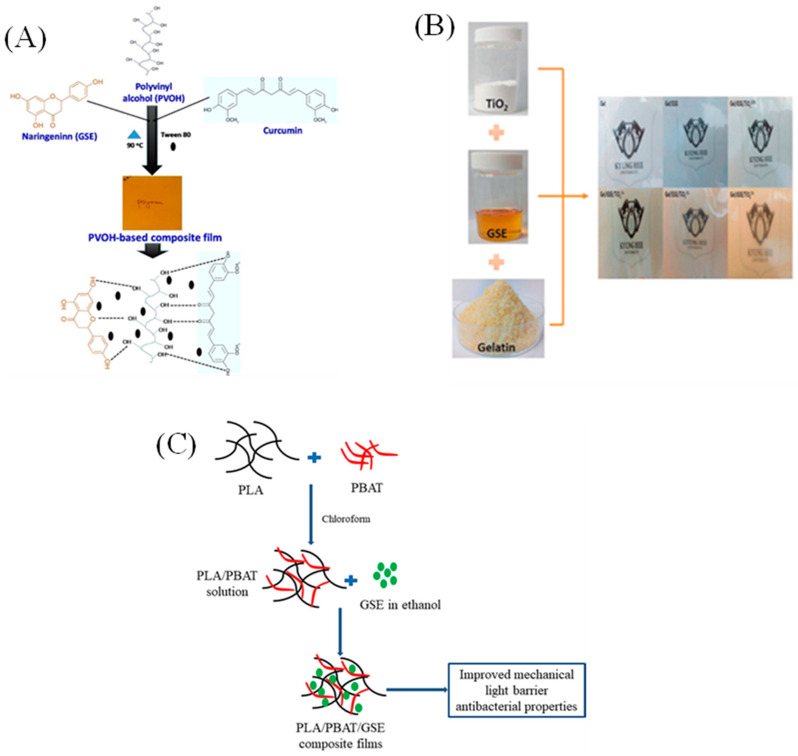
(**A**) Schematic representation of polyvinyl alcohol-based functional composite film added with GSE and curcumin (Reproduced from Roy & Rhim, 2021 [25]); (**B**) Schematic drawing of gelatin-based film incorporated with GSE and TiO_2_ (Reproduced from Riahi et al., 2021 [51]); (**C**) Schematic diagram of polylactic acid (PLA)/poly(butylene-adipate-co-terephthalate) (PBAT)-based composite films mixed with GSE (Reproduced from Shankar & Rhim, 2018 [28]).

The fabrication of GSE-added bio-based packaging films is schematically illustrated in Figure 3. The growing demand for GSE relies on its strong antibacterial and antioxidant activity against a wide range of foodborne pathogens [32,49,50]. The GSE-added film or coating formulation has shown promising results in improving the food shelf-life during storage [30,33]. Moreover, the addition of GSE alone or in combination with bioactive fillers greatly impacted the physical performance of the films [16,19,23,25]. Table 1 shows recent reports on GSE-added food packaging films and the effect of GSE on key physical and functional properties.

## 5. Physical and Functional Properties of GSE- Added Film

The film-forming properties of films containing GSE have been well studied. GSE has high water solubility, producing films/coatings that are well compatible with hydrophilic polymer matrices. In addition, GSE has potent antibacterial and antioxidant activity that imparts functional properties to polymer matrices. Furthermore, since the addition of GSE causes some changes in the physical properties of the films, this section briefly describes the mechanical, barrier, thermal, hydrodynamic, optical, and functional properties of various biopolymer films with the addition of GSE.

### 5.1. Mechanical Properties

Mechanical properties are one of the main parameters of food packaging films as they affect the film’s flexibility and durability. In general, adding fillers has been shown to improve or decrease the film’s mechanical properties [56,57]. In general, it is known that the addition of GSE reduces the mechanical strength of most biopolymer-based food packaging films. For chitosan-based films, adding just 0.5% GSE reduced the mechanical strength by more than 60% (55 MPa to 21 MPa) and significantly increased the flexibility of the film from ~5% to 56% [23]. The change in mechanical properties is presumed to be due to the lowered intermolecular interaction of chitosan chains in the presence of GSE. In addition, blending 5% GSE into carboxymethyl cellulose (CMC)-based films decreased the mechanical strength by ~10% and increased the film’s flexibility by ~10% [24]. Jha (2020) also reported a decrease in tensile strength (~15%) and an increase in elongation at break (3.5-fold) of starch/chitosan-based films after the addition of GSE (0.5–2%) [26]. The decrease in strength may be due to the presence of many polyphenolic components in GSE, and the improvement in flexibility depends on the plasticizer effect of GSE. It has recently been reported that adding GSE to bioplastic and synthetic polymer-based films does not change or improve mechanical performance. For low-density polyethylene (LDPE) and PLA-based melt-extruded films, the strength of the films was slightly decreased by adding GSE, but the difference was not statistically significant [16]. In another report, the inclusion of GSE in PLA/PBAT-based blend films increased the mechanical strength by ~10–20% and the flexibility of the film by ~3-fold [28]. The authors report that the unfamiliar increase in mechanical strength is probably due to the enhanced interaction between the two-polymer matrix with GSE.

### 5.2. Barrier Properties

Water vapor and gas barrier properties are important parameters for packaging films for certain food preservation applications. The resistance of packaging films to water vapor and gas tolerance can depend on several reasons, such as polymer hydrophobicity, mobility, humidity, etc. [58,59]. In general, fruit packaging films have high vapor and gas barrier properties, so it is expected that the respiration of the fruit during storage creates a low oxygen environment and consequently limits the respiration rate [59,60]. Therefore, this section briefly describes the barrier properties of GSE with various packaging films. Recently, it has been reported that adding 5% GSE to CMC-based films improves the water vapor barrier properties of the films by ~10% [24]. In another report, adding 1.5% GSE significantly reduced the water vapor permeability (WVP) of corn starch/chitosan film (70.2 × 10^−11^ g Pa^−1^ s ^−1^ m^−1^ to 5.29 × 10^−11^ g Pa^−1^ s ^−1^ m^−1^), indicating that the water vapor barrier properties of the film were greatly improved [26]. The improvement in the vapor barrier properties of the film can be attributed to the increase in compactness and, consequently, the decrease in intermolecular space.

Conversely, the effect of adding GSE was found to decrease the water vapor barrier properties of carrageenan-based films [19]. In addition, the decrease in water vapor barrier properties was more pronounced for PLA/PBAT and LDPE-based films. Previous reports have shown that the water vapor barrier properties of LDPE-based films are significantly reduced for PLA and LDPE-based films with GSE and PLA/PBAT films [16,28]. Therefore, from the above discussion, it can be concluded that the effects of GSE on packaging polymers are mixed and that the type and properties of each polymer type sometimes improve or even degrade vapor barrier performance.

### 5.3. Thermal Properties

The thermal stability of packaging films is important due to the importance of temperature resistance phenomena during packaging or post-packaging processes. In general, the thermal properties of films are measured using thermogravimetry and differential scanning calorimetry. In general, adding GSE to the polymer matrix for food packaging did not significantly change the thermal properties of the film. In a recent report, the presence of GSE in CMC-based film did not influence the thermal degradation pattern of the film [24]. The addition of GSE increased the final char residue, possibly due to increased heat-stable non-combustible material in the film. In another report, adding 1.5% GSE for starch/chitosan-based improved the thermal stability of the film due to increased intermolecular interaction between polymer and filler [26].

On the other hand, adding 5% GSE in pectin/agar-based binary composite film showed slightly lower thermal stability [52]. The addition of GSE to the gelatin-based film did not show any significant change in the thermal decomposition pattern, except for a slight change in the material after decomposition [51]. For bioplastics (PLA/PBAT-based films), it has been observed that the incorporation of GSE slightly improved the starting temperature of the mixed film, resulting in improved thermal stability [28]. Therefore, the effect of GSE on the thermal stability of the packaging film is inconsistent and can be caused by the properties and properties of the polymer used, the degree of mixing of the polymer with the GSE, etc. 

### 5.4. Water Solubility and Swelling Properties

The film’s water solubility and swelling rate are also important aspects of food packaging. Bioplastic-based films are generally insoluble in water, whereas biopolymer-based films tend to dissolve quickly, limiting their use as packaging materials. Even though biopolymers show high water solubility, in some applications, high water solubility can still be required where the film should be melted before use [60,61]. The mixing of GSE in the packaging matrix showed some impact on the packaging film’s water solubility and swelling degree. Recently, it has been reported that the addition of GSE lowers the water solubility and increases the swelling rate of the pectin/agar-based film [52]. The film’s water activity could be due to the rise in polymer network interaction in the presence of bioactive functional fillers. GSE-added cellulose nanofiber-based films have also increased the swelling rate [50]. The addition of GSE to LDPE and PLA films also showed a decrease in water solubility of these two types of extruded films due to the high hydrophilicity of GSE [16]. GSE’s hydrophilicity is expected to increase the water solubility of the packaging polymer matrix slightly.

### 5.5. Optical Properties

The optical properties of the film are important as it is the apparent appearance of the film, which is very useful for its acceptance by the consumer. The optical properties of a film provide information about its color, transparency, and UV protection properties. The surface color of a film is generally measured using a colorimeter, and film transparency and UV-light barrier properties can be measured through UV-vis spectroscopy analysis [62]. The addition of GSE to the packaging polymer matrix has been shown to affect the film’s optical behavior [22] strongly. In general, high-transparency and colorless films are suitable for food packaging, but adding fillers often changes the color and clarity of the film. The addition of GSE to the polyvinyl alcohol-based film matrix showed a slight increase in yellowness but decreased the transparency of the film [25].

Moreover, the presence of GSE showed some UV-light shielding properties in the film, which may be due to the polyphenol component present in the GSE. In the case of a chitosan-based film, it was reported that the transparency of the film did not decrease even when GSE was added [23]. Similar increases in UV protection properties have been reported for GSE-functionalized gelatin-based films without compromising the transparency of the films [51].

On the other hand, in the case of the PLA/PBAT-based film containing GSE, the transparency of the film decreased sharply, although the UV protection properties were greatly improved [28]. The reduced transparency of the film may be due to improper mixing of the hydrophilic GSE and the hydrophobic PLA/PBAT mixture. Overall, the inclusion of GSE gave the film a bright yellow color, did not significantly alter the transparency of the packaging film, and further showed an improvement in UV shading performance, which could be very useful for food packaging applications. 

### 5.6. Antimicrobial Properties

Antimicrobial activity is essential for functional food packaging films or coatings to prevent unwanted microbial growth and food spoilage during transportation, storage, and packaging. The antimicrobial activity of antibacterial substances or functional packaging films can be measured by disc diffusion, well diffusion, total viable colony count, broth dilution, time kill analysis, etc. The antimicrobial activity of GSE is a major reason for its potential use in food packaging applications. The antibacterial properties of GSE result from the presence of bioactive ingredients, such as naringin, limonin, etc., in GSE [1,63]. The mechanism of antimicrobial activity is still not completely elucidated. It is thought that combinations of polyphenols, like naringin and limonin, enter the microbes’ cell membranes, bind to cellular proteins, disable their function, and subsequently, prevent their growth [19,25]. GSE is added to the food surface directly or as a film/coating to restrict the growth of food spoilage bacteria. The antimicrobial activity of GSE has been tested against varieties of food spoilage pathogens found in fish, meat, fruits, and vegetables [1,63].

There are many reports on the antimicrobial activity of food packaging films or coating formulations. Tan et al. (2015) reported the antifungal activity of the GSE-included chitosan-based packaging film [23]. They reported that the bread packaged with the functional film delayed the mold growth by about 7 days, so it was very effective in improving the shelf life of the bread. The antifungal activity of GSE against *Aspergillus niger* was also observed on chitosan-based films [26]. The author reported that the antifungal activity of GSE increased with increasing content of GSE. In addition, bread wrapped with a functional film was very effective in retarding mold growth in bread specimens, and bread slices packed with 1.5% GSE with chitosan film can delay mold growth by up to 3 weeks. Another paper reported strong antibacterial activity for GSE-added CMC-based packaging films [24]. The addition of 5% of GSE completely restricted the growth of *L. monocytogenes* and *E. coli*. Roy & Rhim (2021) [53] found that the CMC/agar-based film incorporated with 5% GSE completely inhibited the growth of *L. monocytogenes* after 3 h and completely stopped the growth of *E. coli* after 12 h of incubation. Another report also showed that for PLA/PBAT-based films, 7% GSE-added films completely inhibited the growth of *L. monocytogenes* while having a bacteriostatic effect on *E. coli* [28]. In general, the antibacterial activity of GSE was higher in Gram-positive bacteria than in Gram-negative bacteria. The strong antibacterial activity of GSE blended films can effectively control the growth of foodborne pathogens during food packaging. 

### 5.7. Antioxidant Properties

Antioxidant properties are very important for improving the shelf life of packaged foods. The presence of antioxidants in packaging systems generally delays the oxidation and spoilage of foods, extending the shelf life of packaged foods. The antioxidant activity of bioactive compounds or functional packaging films is usually analyzed using DPPH, ABTS, FRAP methods, and cupric reducing, total phenolic content assays. In contrast, oxidation in food is confirmed by lipid peroxide and thiobarbituric acid assays [64,65]. The antioxidant activity of GSE is well known, and its activity is mainly derived from polyphenols such as flavonoids, tocopherols, and other bioactive functional components present in GSE [66]. When GSE was added to the packaging film, the packaging film also showed excellent antioxidant activity. 

Although GSE imparts strong antioxidant activity to the packaging matrix in terms of film or coating, studies on the antioxidant activity of GSE-based packaging films have not been studied much so far compared to the high antibacterial activity of functional films containing GSE. It was recently reported that incorporating GSE into polyvinyl alcohol-based films exhibited potent antioxidant activity determined by the DPPH and ABTS assays [25]. GSE-loaded gelatin-based films also showed potent antioxidant activity in ABTS and DPPH assays [52]. Incorporating GSE in CMC/agar-based and cellulose nanofiber-based films has exhibited moderate antioxidant activity [50,53]. Therefore, the high antioxidant activity of GSE functionalized films is useful for food packaging applications susceptible to photooxidation. 

## 6. Food Packaging Applications of GSE-Incorporated Film and Coating

GSE has a broad antibacterial spectrum against various microorganisms (*Escherichia coli, Candida albicans, Pseudomonas aeruginosa, Clostridium perfringens, Staphylococcus aureus, Salmonella Typhimurium*, and *Listeria monocytogenes*) in real foods. The direct addition of GSE can inhibit the growth of foodborne pathogens in various fruit, vegetable, meat, and fish products, thereby extending the shelf life of foods [1]. Recent progress on GSE-added food packaging films/coatings used in food preservation is summarized in Table 2. As a food preservation technology that recently received much attention from researchers, edible films and coatings are making remarkable achievements in the practical use of various food preservation [67,68,69,70,71,72,73]. Composite edible coatings containing natural plant polyphenols have been used for various food preservation applications, among which GSE, a natural antibacterial agent, is an excellent choice for the manufacture of edible coatings and films [1,15,33,74,75]. 

**Figure 4:**
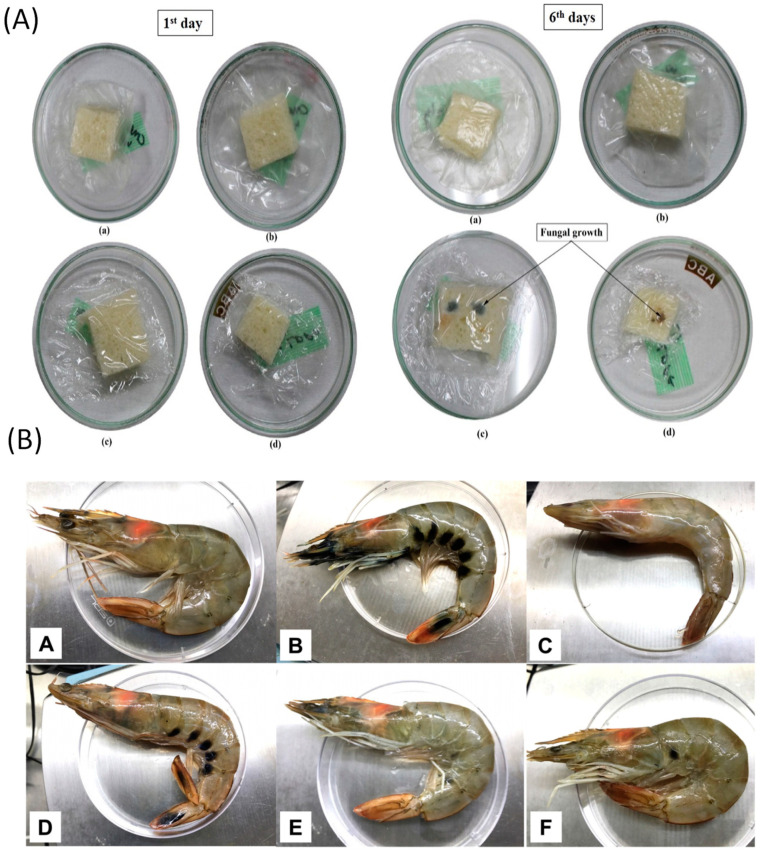
The packaging test of GSE included corn starch/chitosan-based film and alginate/chitosan-based coating. (**A**) Bread sample packed with (a,b) corn starch/chitosan/nanoclay/1.5%GSE films (c,d) low-density polyethylene films. (Reproduced from Jha 2020 [26]), (**B**) Appearance of shrimp during packaging test. Fresh shrimp for untreated control shrimp before and after storage (A–C) and coated with functional film before and after storage (D–F) (Reproduced from Kim et al., 2018 [79]).

Alginate coating has been reported as being effective in weight loss, maintaining firmness, and delaying aging while storing grape berries. The addition of GSE has been reported to maintain antioxidant activity and reduce the incidence of spoilage upon inoculation of treated grape berries [30]. In the case of Redglobe table grapes, treatment with GSE and chitosan inhibited fruit spoilage more effectively than treatment with control fruit and chitosan alone, suggesting that GSE and chitosan had a synergistic inhibitory effect on postharvest fungi [81]. In addition, in a recent study, a chitosan coating containing various concentrations of GSE was developed and applied to cherry tomatoes to protect against the invasion of Salmonella and improve storage properties. It effectively inhibited the growth of Salmonella bacteria and total mesophilic aerobic bacteria with or without GSE. Chitosan coating using GSE was more effective in delaying microbial growth and reducing fruit weight loss than chitosan coating alone. Chitosan/GSE coating did not affect fruit lycopene concentration, color, and organoleptic properties [33]. In another study, corn starch/chitosan films containing GSE were found to be effective in inhibiting fungal growth during bread storage, extending the shelf life of bread and demonstrating more effective food preservation capacity than commercial polyethylene films (Figure 4A) [26]. Edible coatings containing GSE preserve fruits, bread, and other foods. For example, layer-by-layer edible coating treatment of chitosan and sodium alginate containing GSE showed significant inhibition of bacterial growth and maintained low total volatile base nitrogen values in shrimp during storage at 4 °C (Figure 4B) [79]. 

Baek et al. (2021) [76] also developed an alginic acid edible coating containing GSE for shrimp preservation. Compared to the alginate-coated treatment alone, the alginate-coated shrimp with GSE showed lower microbial counts, weight loss, and lower total volatile base nitrogen values during storage. Biodegradable food packaging films containing GSE have shown excellent potential for food preservation. Unlike edible coatings, biodegradable food packaging films are independently produced for packaging use. Hong, Lim, & Song (2009) [78] prepared edible *Gelidium corneum*/gelatin (GCG) blend films containing GSE and used them to test the quality of pork loin meat packed with the films during storage. As the concentration of GSE added to the film increased, the number of microorganisms in packaged pork during storage decreased, and the lipid oxidation level at the end of storage was significantly lower in pork packaged in GSE-containing GCG film than in pork packaged in the neat GCG film. 

Application of edible rapeseed protein/gelatin (RG) film with GSE for packaging Maehyang strawberries reduced the total number of aerobic bacteria, yeasts, and molds in strawberries by 1.03 and 1.34 log CFU g^−1^, and sensory evaluation of strawberries packaged with GSE-RG film showed better sensory scores than the control fruit, in color, appearance, firmness, and odor [32]. In salmon packed in different packages after 15 days of cold storage, *E. coli* O157: H7 and *Listeria monocytogenes* counts were reduced by 0.53 and 0.50 log CFU/g, respectively, compared to unpackaged salmon. In addition, packing salmon in barley bran protein/gelatin films containing GSE reduced peroxide values and thiobarbituric acid values by 23.0% and 23.4%, respectively [34]. Similarly, in another study, it was observed that bread wrapped with chitosan film containing GSE had better quality during storage than bread wrapped with polyethylene and chitosan film, mainly due to the synergistic antifungal effect of GSE and chitosan [23]. Therefore, GSE, as a natural food preservative, improves food preservation capacity when added to edible coatings/films.

## 7. Safety Aspects and Environmental Impact

Pure GSE is non-toxic and chemical-free, making it safe and environmentally friendly. GSE is considered to be a powerful antibacterial and antioxidant of natural origin. Therefore, including pure GSE in food matrices is not expected to harm consumers and the environment. In addition, the bio-based GSE-added packaging is completely safe for the environment. It has recently been reported that some commercial GSEs contain chemicals such as benzalkonium and benzethonium compounds believed to arise from the thermal extraction process of grapefruit seeds using glycerol [1]. As benzalkonium and benzethonium compounds are also known to exhibit potent antimicrobial activity [63], potent antimicrobial activity GSE is thought to be derived from these compounds rather than polyphenols. As benzalkonium and benzethonium compounds exhibit some toxicity at high concentrations [83,84], it is important to confirm the absence of these compounds in the extracted GSE. Pure GSE appears safe for direct or indirect use in food preservation, but further research is needed. Therefore, in conclusion, GSE in its pure form is safe for practical use as a natural preservative in food and food packaging applications.

## 8. Conclusions and Future Perspectives

The use of functional packaging is a promising field that has been growing in recent years. Functional packaging contains bioactive functional compounds, which protect and preserve food from external and internal factors that cause food spoilage. Its strong antibacterial and antioxidant properties make it an ideal bioactive filler for functional packaging due to its non-toxic properties. GSE is applied directly to the food surface to extend the shelf life of the food as well as create a packaging film or coating on the polymer matrix. Adding GSE to polymer matrices can improve food packaging films’ physicochemical and functional properties. Films or coatings with GSE added significantly improved various foods such as fruits, vegetables, fish, meat, and bread.

In conclusion, it is judged that the potential of GSE as a functional material for sustainable food packaging is very high. However, while applying GSE in the food sector looks promising, some challenges remain regarding commercially available GSE. There are some uncertainties related to the natural antimicrobial activity of GSE and some issues related to the commercial manufacture of GSE. Therefore, it is important to identify the exact functional component of GSE responsible for its antimicrobial action. Above all, developing a method for mass-producing GSE-added films for commercial use is essential. Further research into this work will also develop the production of films and coatings, focusing on optimal packaging configurations.

## Figures and Tables

**Table 1:** Overview of the GSE application in the food packaging films.

Polymer Matrix	Added Functional Materials	Properties	References
Carrageenan	GSE	The mechanical strength and water vapor barrier properties decreased while the film’s hydrophilicity and flexibility enhanced. Additionally, the film showed strong antimicrobial activity.	[19]
Chitosan	GSE	Decreased tensile strength and increased flexibility. The antifungal activity of the film was strong enough to defeat the proliferation of fungal growth in bread.	[23]
Pectin/agar	GSE/melanin nanoparticles	Better mechanical and water vapor barrier properties and improved UV-barrier properties of the film. Strong Antibacterial activity toward foodborne pathogens	[52]
Carboxymethylcellulose	GSE/chitin nanocrystal	Reduced mechanical properties but improved UV-barrier properties and Antibacterial activity of the functional film	[24]
Starch-chitosan	GSE/nanoclay	Improved mechanical strength and water vapor barrier properties while reducing the water solubility of the film. The antifungal film was effective in improving the shelf life of bread by up to two weeks	[26]
Poly(lactide)/poly(butylene adipate-co-terephthalate)	GSE	Strong UV-light shielding, better mechanical performance of the film. Antibacterial activity against *L. monocytogenes* but bacteriostatic effect towards *E. coli*	[28]
Gelatin	GSE/TiO_2_	Improved UV-light barrier properties and strong antibacterial activity of the film observed against *L. monocytogenes* and *E. coli*. The thermal stability and mechanical properties were not much affected	[51]
Barley bran protein and gelatin-based film	GSE	The GSE-added film showed strong antibacterial activity toward foodborne pathogenic bacteria, and the film-packed salmon showed less lipid peroxidation	[34]
Cellulose/agar	GSE/alizarin	Enhanced water vapor barrier and UV-light barrier properties of the film. Potent antioxidant and antibacterial activities	[53]
Poly(vinyl alcohol)	GSE/curcumin	Better UV-light barrier and mechanical properties but reduced water vapor barrier properties of the film. Strong antimicrobial activity against foodborne pathogenic bacteria	[25]
Persimmon peel/red algae	GSE	Reduced mechanical and water vapor barrier properties of the film. Strong antibacterial activity against *L. monocytogenes* and moderate activity against *E. coli*	[54]
Cellulose nanofiber	Solution casting	Improved the film’s water vapor barrier and UV-barrier properties without changing mechanical and thermal properties. Strong antibacterial activity against *E. coli* and *L. monocytogenes*	[50]
Corn starch/chitosan	GSE/lemon essential oil	Enhanced antioxidant activity and antibacterial activity toward molds and foodborne pathogenic bacteria	[55]

**Table 2:** GSE-incorporated films/coatings for food packaging applications.

GSE-Added Film/Coatings	Type of Model Food Product for Preservation	References
Rapeseed protein/gelatin films	Strawberry	[32]
Alginate coatings	Grapes	[30]
Red algae film	Cheese and bacon	[29]
Sodium alginate-based edible coating	Shrimp	[76]
Chitosan-based coating	Cherry tomato	[33]
Barley protein-based film	Mushroom	[77]
Gelidium corneum and gelatin-based film	Pork loins	[78]
Alginate and chitosan-based multi-layered coating	Shrimp	[79]
LDPE and PLA-based film	Fish cake	[16]
Coated paper of alginate, carboxymethyl cellulose, and carrageenan	Fish paste	[80]
Chitosan-based coatings	Red grapes	[81]
poly(ε-caprolactone)/chitosan film	Bread	[27]
Poly(lactide)/poly(butylene adipate-co-terephthalate)	Fresh-cut onion, cabbage, and carrot	[82]
Barley bran protein/gelatin	Salmon	[34]

## Data Availability

Not applicable.
